# An efficient method for protein function annotation based on multilayer protein networks

**DOI:** 10.1186/s40246-016-0087-x

**Published:** 2016-09-27

**Authors:** Bihai Zhao, Sai Hu, Xueyong Li, Fan Zhang, Qinglong Tian, Wenyin Ni

**Affiliations:** Department of Mathematics and Computing Science, Changsha University, Changsha, Hunan 410022 China

## Abstract

**Background:**

Accurate annotation of protein functions is still a big challenge for understanding life in the post-genomic era. Many computational methods based on protein-protein interaction (PPI) networks have been proposed to predict the function of proteins. However, the precision of these predictions still needs to be improved, due to the incompletion and noise in PPI networks. Integrating network topology and biological information could improve the accuracy of protein function prediction and may also lead to the discovery of multiple interaction types between proteins. Current algorithms generate a single network, which is archived using a weighted sum of all types of protein interactions.

**Method:**

The influences of different types of interactions on the prediction of protein functions are not the same. To address this, we construct multilayer protein networks (MPN) by integrating PPI networks, the domain of proteins, and information on protein complexes. In the MPN, there is more than one type of connections between pairwise proteins. Different types of connections reflect different roles and importance in protein function prediction. Based on the MPN, we propose a new protein function prediction method, named function prediction based on multilayer protein networks (FP-MPN). Given an un-annotated protein, the FP-MPN method visits each layer of the MPN in turn and generates a set of candidate neighbors with known functions. A set of predicted functions for the testing protein is then formed and all of these functions are scored and sorted. Each layer plays different importance on the prediction of protein functions. A number of top-ranking functions are selected to annotate the unknown protein.

**Conclusions:**

The method proposed in this paper was a better predictor when used on *Saccharomyces cerevisiae* protein data than other function prediction methods previously used. The proposed FP-MPN method takes different roles of connections in protein function prediction into account to reduce the artificial noise by introducing biological information.

**Electronic supplementary material:**

The online version of this article (doi:10.1186/s40246-016-0087-x) contains supplementary material, which is available to authorized users.

## Background

The accurate annotation of protein functions is the key to understanding life at the molecular level and has great biomedical and pharmaceutical implications. Due to high-throughput biological technologies, a large number of protein sequences [1] are available, while majority of their functions are still unknown. With its inherent difficulty and expense, experimental characterization of protein functions cannot accommodate the ever-increasing number of sequences and structures produced by Genomics Centers. Recent developments in experiments such as yeast two-hybrid [2], tandem affinity purification [3] and mass spectrometry [4] have resulted in the publications of many high-quality, large-scale protein-protein interaction (PPI) data, which make it possible and feasible to use computational methods to predict functions for un-annotated proteins [5].

The past decade has witnessed a rapid development of computational methods for predicting protein functions from PPI datasets. A neighbor counting (NC) method proposed by Schwikowski et al. [6] predicted an un-annotated protein with the functions that occurred most frequently among its neighbor proteins. However, this method ignored the background frequency of different function annotations. Hishigaki et al. [7] improved the neighbor counting method by using the Chi-Square statistics instead of frequency as a scoring function. Besides direct neighbors, Chua et al. [8] inferred the functional information within both direct (level 1) and indirect (level 2) neighbors by giving them different weights. Prior methods typically measured proximity as the shortest-path distance in the network, while most proteins are close to each other. Cao et al. [9] introduced diffusion state distance (DSD), a new metric based on a graph diffusion property, designed to capture finer-grained distinctions in proximity for transferring functional annotation in PPI networks. Other methods have been introduced to make functional prediction by getting the most consistent agreement throughout the whole PPI networks [10]. Chi et al. [11] proposed an approach that predicted protein functions iteratively. This iterative approach incorporated the local and global semantic influence of protein functions into the prediction. Some kind of network-based methods partitioned proteins in PPI networks into several function modules [12], and the proteins in the same modules are assigned with the same functions. Lee et al. [13] applied a novel method that generated improved modularity solutions, and developed a better method to use this community information to predict protein’s functions.

Taking both high noise in PPI data and insufficient number of available annotated proteins into account, some researchers have tried to improve the prediction performance by incorporating other heterogeneous data sources. Cozzetto et al. [14] proposed an integrative approach for addressing annotation challenge, which combines into a wide variety of biological information sources encompassing sequence, gene expression, and PPI data. Zhang et al. [15] presented a novel protein function prediction method that combined protein domain composition information and PPI networks. Domain combination similarity (DCS) [16] was applied to predict protein function by integrating PPI networks and proteins’ domain information. Different from Zhang’s, DCS changed the method to calculate domain context similarity and combined the domain compositions of both proteins and their neighbors. Liang et al. [17] built a network model called protein overlap network (PON) using domain co-occurrence information. In a PON, each node represented a protein and two nodes were connected with an edge if they share a common domain. The function of a protein can be predicted by counting the occurrence frequency of gene ontology (GO) terms associated with domains of direct neighbors in the PON. Recently, some new algorithms are proposed to predict protein function from PPI networks. Gong et al. [18] developed a method named GoFDR for predicting GO-based protein functions. The input for GoFDR is simply a query sequence-based multiple sequence alignment (MSA) produced by PSI-BLAST (Position-Specific Iterated BLAST). Kumar et al. [19] proposed an improved approach for protein function prediction by exploiting the connectivity properties of prominent proteins. Yu et al. [20] proposed a method called Predicting Protein Function using Multiple Kernels (ProMK). ProMK iteratively optimizes the phases of learning optimal weights and reduces the empirical loss of multi-label classifier for each of the labels simultaneously.

In conclusion, many computational methods that integrate heterogeneous data for predicting protein (or gene) functions have been suggested. Most of these techniques follow the same basic paradigm: firstly, they generate various functional association networks by analyzing implicit information of shared functions of proteins from different data sources. Then these individual networks are combined into a composite and highly reliable network through a weighted sum. The weight of each individual network represents the contribution of the corresponding data source to the function prediction. A correct setting of these weights is thought to be the key to designing an effective function prediction method. In general, the weights adjustment of individual networks is mainly influenced by human experience and statistical analysis. The major drawback of how each network is weighted is that it varies between different datasets. Furthermore, functions of proteins are diverse and some of them only occur under specific conditions. Different functional association networks play different roles and have varying importance in function prediction. Combining a heterogeneous data source into a single weighted network could obscure the inherent nature of the protein function.

To address these difficulties, we construct a multilayer protein network which integrates PPI network topology, domain information, and protein complexes. Additionally, we propose an efficient protein function annotation method, named FP-MPN (function prediction based on multilayer protein networks). FP-MPN takes into account the varying influences by multiple connections in the prediction of protein function. Given an un-annotated protein, FP-MPN generates candidate functions by examining multilayer networks systematically in turn. The performance of FP-MPN was tested on the well-studied species of *Saccharomyces cerevisiae*. Compared to several previously reported protein function prediction algorithms, FP-MPN achieved a greater degree of accuracy in predicting protein function. The experimental results demonstrate that this method, which distinguishes different types of connections in function prediction, is more robust and effective than those methods combining multiple interactions, and that FP-MPN is a good example of this.

## Materials and methods

### Assessment criteria

Cross-validation is a widely used method to evaluate the performance of protein function prediction algorithms. The proteins in the PPI network are partitioned into two subsets, the training set and the testing set. Functions are removed from the part of proteins in the PPI network artificially. These proteins consist of the testing set and the rest proteins form the training set. Functions of proteins in the testing set are predicted, using functional information of proteins in the training set. Finally, the comparing results of predicted functions with actual functions are used to evaluate the performance of protein function prediction algorithms. The cross-validation methods can be classified into two categories: leave-one-out cross-validation and leave-percent-out cross-validation. The leave-one-out cross-validation method puts one protein into the testing set and the remaining proteins into the training set, while the leave-percent-out cross-validation method randomly selects a percentage of proteins as the testing set and then puts other proteins into the training set. Each function of proteins in the testing set is assigned with a probability, according to the functions of proteins in the training set. Then a number of top-ranking functions are selected to annotate the protein with unknown functions. The quality of prediction depends on the matching results of predicted functions with actual ones. There are two widely used criteria to measure the predicted results. The one is Precision which measures the percentage of predicted functions that match the known functions. The other is Recall which measures the fraction of known functions that are matched by the predicted ones. They can be calculated as follows:

1 $$ \mathrm{Precision}=\frac{\mathrm{TP}}{\mathrm{TP} + \mathrm{F}\mathrm{P}} $$

2 $$ \mathrm{Recall}=\frac{\mathrm{TP}}{\mathrm{TP} + \mathrm{F}\mathrm{N}} $$

where TP (true positive) is the number of predicted functions matched by known functions. FP (false positive) is the number of predicted functions that are not matched by known functions. FN (false negative) is the number of known functions that are not matched by predicted functions. Selecting more functions can improve the recall, but it may lead to the reduction of precision. *F*-measure, as the harmonic mean of precision and recall, is another measure to evaluate the performance of a method synthetically, which is calculated as follows:

3 $$ F\hbox{-} \mathrm{measure}=\frac{2 \times \mathrm{Precision} \times \mathrm{Recall}}{\mathrm{Precision} + \mathrm{Recall}} $$

At the same time, the coverage rate (CR) [21] is also used to evaluate a function prediction algorithm, which shows how many functions of proteins in the testing set can be covered by predicted functions. Given a testing protein set TP = {tp_1_, tp_2_, …, tp_*n*_}, KF = {kf_11_, kf_12_,…, kf_*ij*_, …, kf_*nm*_} is a list of known function sets of TP, KF_*i*_ = {kf_*i*1_, kf_*i*2_,…, kf_*il*_} is a known function set of the protein tp_*i*_. PF = {pf_11_, pf_12_,…, pf_*ij*_, …, pf_*nm’*_} is a list of predicted function sets of TP, PF_*i*_ = {pf_*i*1_, pf_*i*2_,…, pf_*il’*_} is a predicted function set of the protein tp_*i*_. The coverage rate is then defined as

4 $$ \mathrm{C}\mathrm{R}={\displaystyle \sum_{i=1}^n\left|K{F}_i\cap P{F}_i\right|}/{\displaystyle \sum_{i=1}^n\left|K{F}_i\right|} $$

### Motivation

Some methods try to reconstruct more reliable networks by integrating PPI networks and biological information, in order to reduce the impact of random noise on predicting performance. There exist complex and diverse relationships between proteins as demonstrated after integrating biological information. For example, proteins can interact with each other through physical interactions which can be identified by biological experiments, co-expression based on time course gene expression data [22, 23], or co-annotation based on gene ontology [24, 25], etc. Most of these methods generate various functional association networks, such as co-expression networks and co-annotation networks. Then a single network can be constructed through a weighted sum of these individual networks. The weight assigned to each individual network reflects its contribution towards protein function annotation, which is computed by a specific similar metric for the related biological data.

**Fig. 1:**
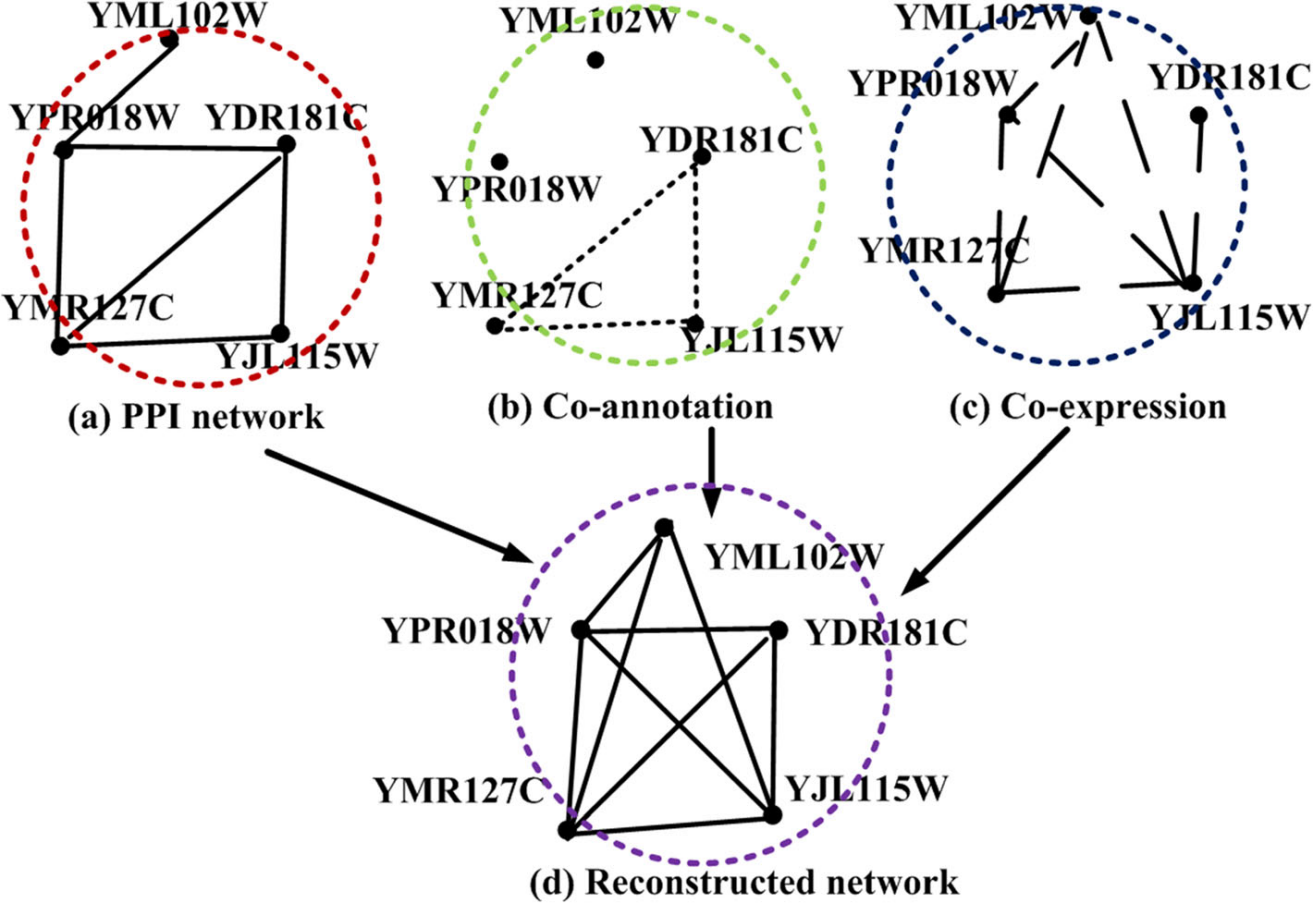
**a** is the original protein-protein interaction network experimentally validated. **b** is the constructed co- annotation network based on the GO profile. **c** is the constructed co-expression network based on time course gene expression data. **d** is reconstructed network based on the PPI network, co- annotation network and co-expression network by current methods

Figure 1 describes an example of constructed networks by integrating the PPI network and heterogeneous data. Figure 1a shows an original physical PPI network, which was derived from experimental methods. In the co-annotation network, as shown in Fig. 1b, there exists a connection between a pair of proteins if they perform the same functions. As for the co-expressed network, it is based on time course gene expression data. For a protein *v*, its gene expression at *n* different times is denoted as a variate:*Gen*(*v*) = {*T*(*v*, 1), *T*(*v*, 2), …, *T*(*v*, *n*)}, *T*(*v*, *i*) denotes the expression level of gene *v* at the time point *i*. Generally, the Pearson correlation coefficient [26] is used to assess the probability of whether two particular proteins are co-expressed. If the Pearson correlation coefficient of two proteins over all time points is greater than 0.8, then they are considered to be co-expressed and are connected in the co-expressed network. The network shown in Fig. 1d is a reconstructed network based on three networks currently used. This network shows that proteins could have a diversity of functions when exposed to different conditions or at different time points. Therefore, the importance and roles of different types of interactions between proteins are not the same for the protein function prediction. When functions are predicted for the unknown protein YJL115W using the constructed network in Fig. 1d, YPR018W and YDR181C are treated in the same way. The connection (YPR018W, YJL115W) and (YDR181C, YJL115W) has the same status and reliability (they both have an edge clustering coefficient [27] of one). After analyzing the original PPI network, co-annotation network, and co-expression network as shown in Fig. 1, it is demonstrated that the connection (YDR181C, YJL115W) is more reliable than (YPR018W, YJL115W), due to its occurrence in all three networks. YPR018W and YJL115W are only co-expressed at the gene expression level, based on gene expression data. Therefore, YDR181C should contribute more to the function prediction of YJL115W, than the protein YPR018W. Connections between YDR181C and YJL115W overlap in the reconstructed network; therefore, it is difficult to determine their relationship. The information mentioned above was obtained from the reconstructed network.

The analysis of this experiment suggests that existing methods have two deficiencies. Different biological data sources (i.e., PPI networks, protein domains, and subcellular information) often describe protein properties in different ways and have different correlations with different GO terms. Combining multiple biological data into a single network can not only enhance the matching accuracy (i.e., recall, which measures the fraction of known functions that are matched to the predicted ones) to a certain extent but also introduce a lot of noise functions and reduce predicting accuracy (i.e., precision, which measures the percentage of predicted functions which match the known functions). As a result, the comprehensive performance improvement is not apparent. Current methods set different weights for heterogeneous data based on the quality of data sources in order to integrate them into a single network. Setting the weighting system for multiple biological data is the key to ensuring the accuracy of protein function prediction. These optimal weighting methods rely on empirical analysis and have differences between datasets. Furthermore, these weighting methods may also lead to the inconsistency of these prediction algorithms.

In conclusion, it is inappropriate to combine multiple interactions or connections between two proteins, as they often occur under different conditions and play different roles in protein function prediction. In this paper, we describe a multilayer protein network developed by integrating PPI network topology and heterogeneous data. In the constructed network, a pair of proteins has more than one connection which is connected through multiple links. Based on the multilayer protein network, we propose a new method for predicting protein functions, named FP-MPN.

### Multilayer protein networks construction

The complex network is a hot, new research area as a result of the increased use of networks in various fields, such as mathematics, social science, and life science. The features of many real-life complex networks are that they are small-world (i.e., high clustering coefficient and small average path length) and scale-free (i.e., follow the power-law distributions in node degree and display the growth and preferential attachment). In reality, connections among nodes in complex networks are diversified. For instance, in social networks, people can contact each other via emails, telephones or MSN, etc., and hence make up a complex network with multi-links. Similarly, in biological networks there are diverse links among proteins via co-expression or co-annotation of the proteins. Multilayer networks are more complex than those with single link.

**Fig. 2:**
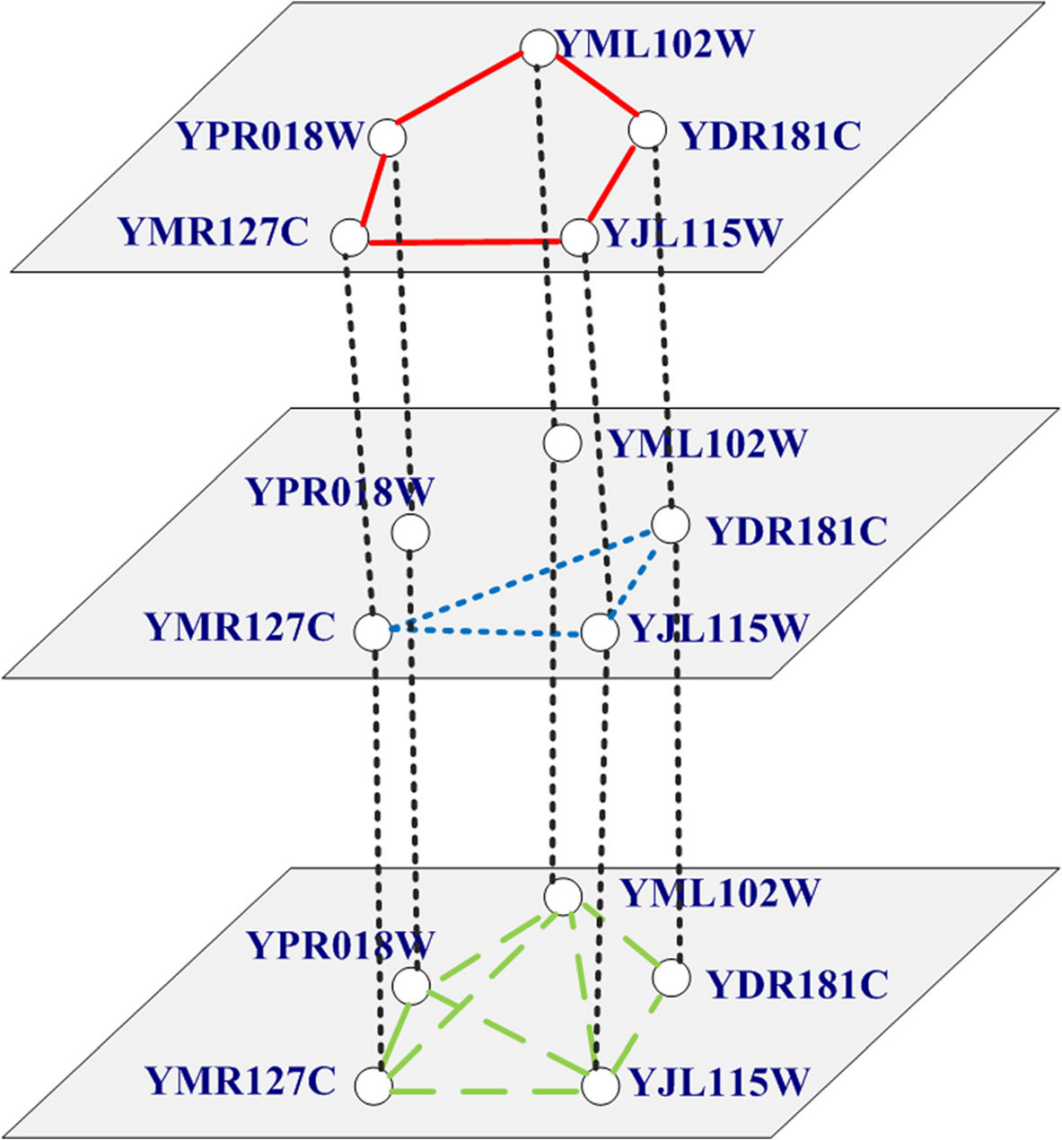
Example of multilayer protein networks

We consider a multilayer network *G* = (*V*, *E*), where *V* = {*v*_1_, *v*_2_,…, *v*_*n*_} represents a set of proteins, the edge set *E* = {*Me*_1_, *Me*_2_,…, *Me*_*m*_} consists of edges of *L* different types representing different relations. That is, *Me*_*i*_ = {*e*_*i*1_, *e*_*i*2_,…, *e*_*iL*_} (0 < *i < =m*), *e*_*ij*_ (0 < *j < =L*) represents the *i*th connection in the *j*th layer of *G*. We can view the multilayer network as a graph with vector valued edge information, i.e., the adjacency matrix *A* consists of elements *A*_*ij*_, who are themselves *L* dimensional vectors: *A*_*ij*_ = {*A*_*ij*_^(1)^, *A*_*ij*_^(2)^,…, *A*_*ij*_^(L)^}. An alternative way to approach the problem is to view the multi-graph as a collection of *L*, *N* × *N* adjacency matrices {*A*^(1)^, *A*^(2)^,…, *A*^(L)^}, each corresponding to one type of relation. Figure 2 describes an example of a multilayer network according to Fig. 1. The multilayer network consists of five nodes and three layers. Each layer represents a different level of connection or relationship between nodes.

Functions are often performed by proteins physically interacting with each other, located within the same complex, or by having similar structures. A protein consists of one or more domains which have independent functions. There may be discrepancies within domain combinations among different proteins and it is of great significance to recognize these. In this paper, we develop a multilayer network by integrating the PPI network, protein domain information, and protein complexes. The multilayer network consists of three layers, which include the physical interaction layer (PIL), sharing domain layer (SDL), and sharing complex layer (SCL). The physical interaction layer is derived from original PPI networks. On the SDL, two proteins are physically connected if there is at least one domain common to both of them. On the SCL, each node represents a protein and two nodes are physically connected if they are contained in a common complex. Our previous research on protein complex prediction [28] and essential protein identification [26] suggests that the performance of the prediction algorithm based on weighted networks is superior to that based on un-weighted networks. An explanation for this could be that the weight stands for the reliability of interactions and therefore, weighted networks can be more useful than un-weighted networks in the representative of PPI networks. In this work, appropriate weighting methods for the three types of connections are developed for the multilayer network.

Methods of Zhang and DCS successfully integrated domain information and PPI networks, improving the performance of protein function prediction. The two methods rely on the same principle, which is to implement function prediction by way of computing similarities between the two proteins. The two methods differ in that the method described by Zhang only computes similarity through the domain information of the protein itself, while the DCS method expands on the extra domain information of the neighbors surrounding it. The two methods are all based on the computing similarity of the combination formula. However, they have the problem of being highly complex to program. To balance the pros and cons of the two methods, this study has set up the weighting computational formula aiming at the interaction of shared domain as follows:

5 $$ W\left({v}_i,{v}_j\right)=\left\{\begin{array}{ccc}\hfill \frac{\left|{D}_i\cap {D}_j\right|{}^2}{\left|{D}_i\left|\times \right|{D}_j\right|}\hfill & \hfill, \hfill & \hfill {D}_i\ne \varnothing\ \mathrm{and}\ {D}_j\ne \varnothing \hfill \\ {}\hfill 0\hfill & \hfill, \hfill & \hfill \mathrm{otherwise}\hfill \end{array}\right. $$

where *D*_*i*_ and *D*_*j*_ are sets of distinct domain types of *v*_*i*_ and *v*_*j*_, respectively.

In a similar way, the weight of sharing complexes between *v*_*i*_ and *v*_*j*_ on the SCL can be calculated as follow:

6 $$ W\left({v}_i,{v}_j\right)=\left\{\begin{array}{ccc}\hfill \frac{\left|{C}_i\cap {C}_j\right|{}^2}{\left|{C}_i\left|\times \right|{C}_j\right|}\hfill & \hfill, \hfill & \hfill {C}_i\ne \varnothing\ \mathrm{and}\ {C}_j\ne \varnothing \hfill \\ {}\hfill 0\hfill & \hfill, \hfill & \hfill \mathrm{otherwise}\hfill \end{array}\right. $$

where *C*_*i*_ and *C*_*j*_ are the sets of protein complexes that contained *v*_*i*_ and *v*_*j*_, respectively, and *C*_*i*_∩*C*_*j*_ denotes the set of common protein complexes.

As for the weight of connections on the PIL, we suggest that the weight of an interaction can be reflected by the number of common neighbors between the proteins. Here we use a variant of edge clustering coefficient (ECC) [27] to calculate the weight of protein pairs. Given a pair of proteins *v*_*i*_ and *v*_*j*_, the weight of edge (*v*_*i*_, *v*_*j*_) on the PIL is defined as follows:

7 $$ W\left({v}_i,{v}_j\right)=\left\{\begin{array}{ccc}\hfill \frac{\left|{N}_i\cap {N}_j\right|{}^2}{\left(\left|{N}_i\right|-1\right)*\left(\left|{N}_j\right|-1\right)}\hfill & \hfill, \hfill & \hfill \left|{N}_i\right|>1\ \mathrm{and}\ \left|{N}_j\right|>1\hfill \\ {}\hfill 0\hfill & \hfill, \hfill & \hfill \mathrm{otherwise}\hfill \end{array}\right. $$

where *N*_*i*_ and *N*_*j*_ are sets consisting of all neighbors of *v*_*i*_ and *v*_*j*_, respectively.

**Fig. 3:**
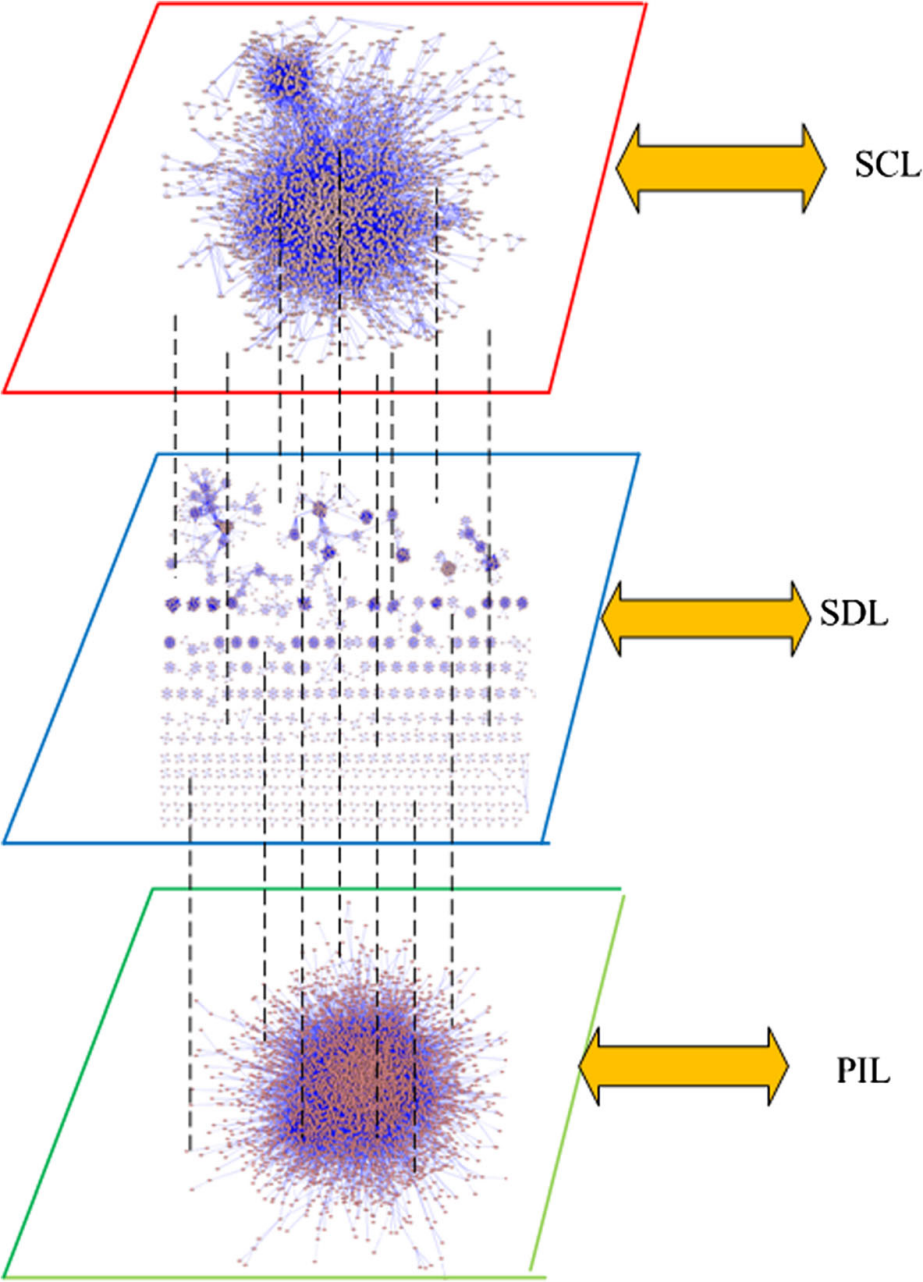
Visualization of constructed multilayer protein networks

Figure 3 is the visualization of our constructed multilayer protein network. The network consists of three layers, i.e., PIL, SDL, and SCL. There are the same set of proteins and different connections sets on these three layers. The multilayer protein network can be modeled as *G* = (*V*, *E*), where *V* = {*v*_1_, *v*_2_,…, *v*_*n*_}, *E* = {*Me*_1_, *Me*_2_,…, *Me*_*m*_}. *Me*_*i*_ = {*e*_*i*1_, *e*_*i*2_, *e*_*i3*_} (0 < *i < =m*), *e*_*ij*_ (0 < *j < =3*) represents the *i*th connection in the *j*th layer of *G*.

### FP-MPN algorithm

Based on the weighted multilayer protein network, we propose a new method for protein functional prediction, named FP-MPN. How to deal with the multilayer networks is the first problem to be addressed. Current algorithms combine different connections into a single connection when dealing with these complex biological networks. In reality, it is inappropriate to combine multiple connections between two proteins, as they often occur under different conditions and play different roles in protein function prediction. The influences of different types of interactions in protein function prediction are not the same. Combining different interactions into a single event can lead to false positive results. So, it is necessary to deal with multilayer networks in another way.

The different connections among proteins may have different impacts on function prediction. To address this, FP-MPN visits each layer of the multilayer network in turn to generate candidate functions. Each layer has different contribution to predict ion of functions for an un-annotated protein. The FP-MPN algorithm operates in two stages, pre-processing data and predicting functions.

**Fig. 4:**
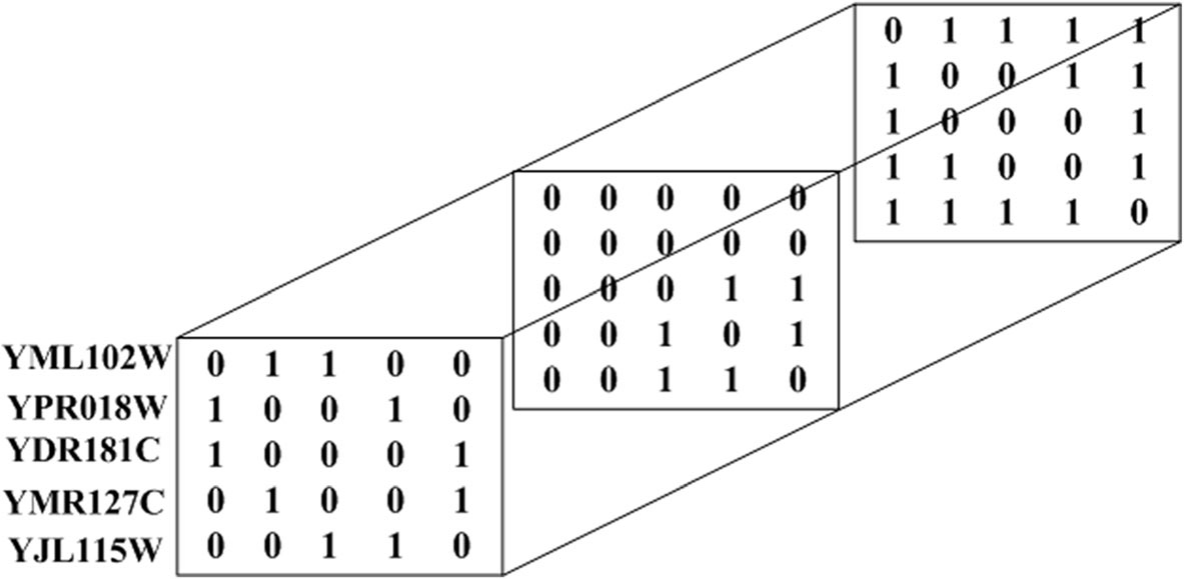
The tensor representation of a multilayer protein network

To assign functions of proteins in the testing of a set of probabilities, pre-processing of the multilayer protein network is required. The constructed multilayer protein network can be represented as a tensor *A* = (*a*_*i,j,k*_) _*n*×*n*×*m*_, where *n* is the number of proteins and *m* is the number of types of interconnections. If node *i* is connected to node *j* by the *k*th type link, *a*_*i,j,k*_ is equal to 1; otherwise, it equals 0. Figure 4 depicts the tensor representation of the multilayer network as shown in Fig. 2. Given a tensor *A*, we can get a new tensor *A*^(1)^, which is calculated as follows:

8 $$ {a}_{i,j,k}^{(1)}=\left\{\begin{array}{ccc}\hfill {a}_{i,j,k}/{\displaystyle \sum_{j=1}^n{a}_{i,j,k}}\hfill & \hfill, \hfill & \hfill {\displaystyle \sum_{j=1}^n{a}_{i,j,k}}>0\hfill \\ {}\hfill 0\hfill & \hfill, \hfill & \hfill \mathrm{otherwise}\hfill \end{array}\right. $$

Therefore, for each row *i* of the tensor *A*^(1)^, ${\displaystyle \sum_{j=1}^n{a}_{i,j,k}^{(1)}}=1$ or ${\displaystyle \sum_{j=1}^n{a}_{i,j,k}^{(1)}}=0$.

The second stage of FP-MPN is predicting functions for un-annotated proteins. The FP-MPN method visits each layer of the corresponding multilayer network of the tensor *A*^(1)^, Given that the proteins interact with each other under different conditions or stimuli in order to perform different functions, FP-MPN generates predicted functions across all layers. While the importance of each layer to the prediction is not the same. We assign different importance coefficient (IC) for each layer of the MPN. For the *i*th layer, its IC value can be calculated as follow:

9 $$ \mathrm{I}\mathrm{C}(i)=\frac{1}{2^i} $$

The final score of a predicted function is the weighed sum of scores achieved from all layers. The IC value of a layer is used to present the weight. The layer accessed firstly has higher IC value than that rest of the layers. For this reason, the set up access sequence of each layer in the MPN is critical for the FP-MPN method. This paper addresses the problem of the impact of each layer on the accuracy of function predictions using statistical analysis. More detailed statistical results can be found in Table 1.

**Table 1:** Statistical analysis of the influence of three layers

Layers	Annotated proteins	Precision	Recall	*F*-measure
PIL	1274	0.3791	0.1094	0.1697
SDL	1215	0.3595	0.1538	0.2154
SCL	1103	0.3404	0.1829	0.238

In this experiment, we used the NC [6] method on the SDL, SCL, and PIL to annotate all unknown proteins, using leave-one-out cross-validation. Then, we calculate the average Precision, Recall, and *F*-measure to evaluate the significance of each layer for function prediction. The original PPI network consisted of 5093 proteins with 24,743 interactions. For the PIL, SDL, and SCL, there are 13,871, 23,749, and 7337 connections, respectively. Using PIL, there are 2388 proteins, which had at least one neighbor. The number of nodes with neighbors on the SDL and SCL is 2972 and 1494, respectively. From Table 1, it can be seen that SCL archives the highest *F*-measure among the three layers. In addition, 73.83 % (1103/1494 = 73.83 %) of proteins with neighbors on the SCL have been annotated as at least one function. While the proportion of PIL and SDL is 53.35 % (1274/2388 = 53.35 %) and 40.88 % (1215/2972 = 40.88 %), respectively. The SDL gets the second highest *F*-measure and Recall after SCL among all the layers. Thus, we assigned the highest access sequence to SCL, the second highest priority to SCL, and the lowest order to PIL.

**Fig. 5:**
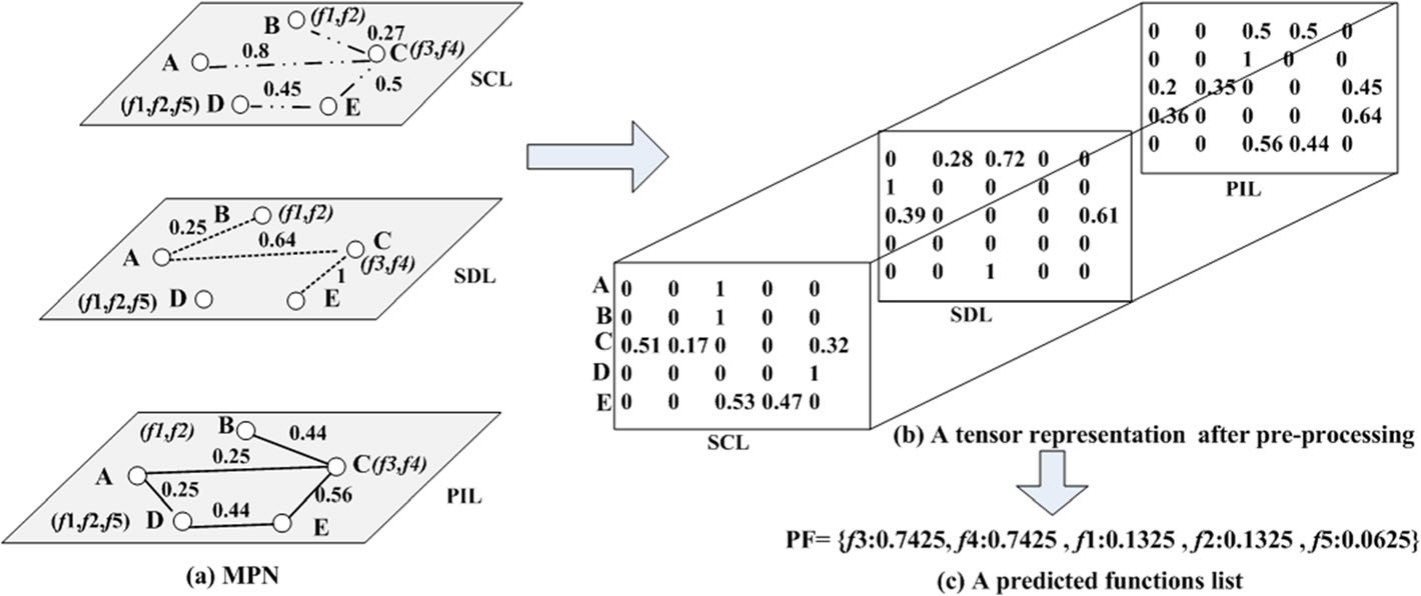
**a** is the constructed multilayer protein network. **b** is the tensor representation of MPN after pre-processing. **c** is the predicted functions list for the un-known protein A generated by the FP-MPN method

The second stage of FP-MPN consists of two major steps. The first step is to search its neighbors in the MPN for a particular protein *u* with unknown function, to generate candidate functions. Starting from the layer in MPN which has the highest access sequence, the FP-MPN method creates a functions list *PF*. These lists of functions are derived from neighbors of the testing protein *u*. Assume that *P* = {*p*_1_, *p*_2_,…, *p*_*n*_} is a set of neighbors of the protein *u* on the first layer, *F* = {*f*_1_, *f*_2_,…, *f*_*m*_} is a set of functions of all these proteins in *P*. The score of a certain function *f*_*j*_ in *F* can be calculated by the following formula:

10 $$ S\left({f}_j\right)={\displaystyle \sum_{i=1}^nW\left(u,{p}_i\right)}\times {t}_{ij},\kern0.5em \left(j\in \left[1,m\right]\right) $$

where *W*(*u*, *p*_*i*_) represents the weight of the connection between *u* and *p*_*i*_. If *p*_*i*_ contains function *f*_*j*_, then *t*_*ij*_ = 1, otherwise *t*_*ij*_ = 0. Then, the FP-MPN enters the next layer of MPN and continues to predict functions. If a function has been predicted on previous layers, its score is accumulated. This process is repeated for the next layer etc., until all the layers are traversed. For a predicted function *f*, its final score is the weighed sum of scores on all layers and can be calculated as follow:

11 $$ \mathrm{Score}(f)={\displaystyle \sum_{i=1}^LIC(i)}*S\left({f}_i\right) $$

where *L* is the number of layers, IC(*i*) is the IC value of the *i*th layer, and *S*(*f*_*i*_) is the score of function *f* on the *i*th layer calculated using Equation (10). From Equation (9), it is not difficult to deduce the formula ${\displaystyle \sum_{i=1}^m\mathrm{I}\mathrm{C}(i)<1}$, thus ensuring that Score(*f*) is less than 1 and can be used as a probability of the function *f*. Figure 5 illustrates how the FP-MPN method gets the predicted functions list. Figure 5a depicts the constructed multilayer protein network. Numbers on the edges of each layer in the MPN represent their corresponding weights. Figure 5b is the tensor representation of MPN after pre-processing, using Equation (8). Figure 5c shows the predicted functions list for the unknown protein *A* generated by the FP-MPN method. In this example, FP-MPN predicts functions *f*3 and *f*4 according to its neighbors on the SCL. FP-MPN computes the scores of *f*3 and *f*4 on the SCL by Equation (10), which is 1 and 1, respectively. Then, FP-MPN enters the SDL and continues to generate functions. The candidate function set of *A*’s neighbors on SDL consists of {*f*1, *f*2, *f*3, *f*4}. The score of *f*1, *f*2, *f*3, *f*4 on the SDL is 0.28, 0.28, 0.72, and 0.72, respectively. In a similar way, FP-MPN records the functions {*f*1, *f*2, *f*3, *f*4, *f*5} on the PIL. Scores of the five functions are the same that is 0.5. According to Equation (11), the final score of *f*3 can be calculated as follow:

$$ \mathrm{Score}\left({f}_3\right)=1*\frac{1}{2}+0.72*\frac{1}{2^2}+0.5*\frac{1}{2^3}=0.7425 $$

The final score of *f*1, *f*2, *f*4, *f*5 is 0.1325, 0.1325, 0.7425, and 0.0625, respectively.

The last step of the second stage is to rank functions according to their scores and select a top *N* of the ranked functions for the protein with unknown function. This is a key factor which influences the performance of the function prediction algorithm. Existing methods for function selection are mainly implemented in two ways: one is represented by the methods of Zhang [15] and DCS [16], which computes the similarity between proteins and endow all functions of the protein with the highest similarity to the protein with unknown function. Another is represented by the method of NC, which forms candidate functions set by all the functions of the neighbors, then grades and ranks these functions according to a strategy. We have performed statistical analysis for the overlap of functions between the annotated proteins, in order to determine a solution to function selection, as shown in Table 2.

**Table 2:** Statistical analysis of overlaps of functions

OS	Proportion (all proteins)	Proportion (proteins with more than one function)
(0, 0.2]	2.81 %	5.64 %
(0.2, 0.4]	13.90 %	27.95 %
(0.4, 0.6]	27.05 %	54.41 %
(0.6, 0.8]	2.02 %	4.06 %
(0.8, 1]	54.22 %	7.93 %

The first column in Table 2 refers to the function overlap between each pair of proteins. The function overlap score of two proteins *u* and *v* is defined as follows [28]:

12 $$ OS\left(u,v\right)=\frac{\left|{F}_u\cap {F}_v\right|{}^2}{\left|{F}_u\left|\times \right|{F}_v\right|} $$

where *F*_*u*_ and *F*_*v*_ is the function set of proteins *u* and *v*, respectively. The second column in Table 2 has shown statistical results of overlaps of all pairs of proteins with shared functions, among which the overlap score of 54.22 % protein pairs has exceeded 0.8. As many proteins have only one function, we made statistics again after excluding those with only one function (the result is shown in the third column). It turned out that the overlap score of more than half of the protein pairs falls in (0.4, 0.6], and the protein pairs with overlap score over 0.6 accounts for only 11.99 %. Based on these statistical results, the FP-MPN method adopts the second strategy of function selection mentioned above.

All functions are sorted in descending order according to their scores. The top *N* of these functions can be selected to annotate the testing protein *u*, where *N* is the number of functions of the protein most closely associated with *u*. In this paper, we used the highest weight of a pair of proteins to evaluate the close degree of all their layers. We limited the number of predicted functions to be less than or equal to that of the annotated GO terms in the protein with highest weight to *u*. Algorithm FP-MPN illustrates the overall framework to predict protein functions based on multilayer protein networks.
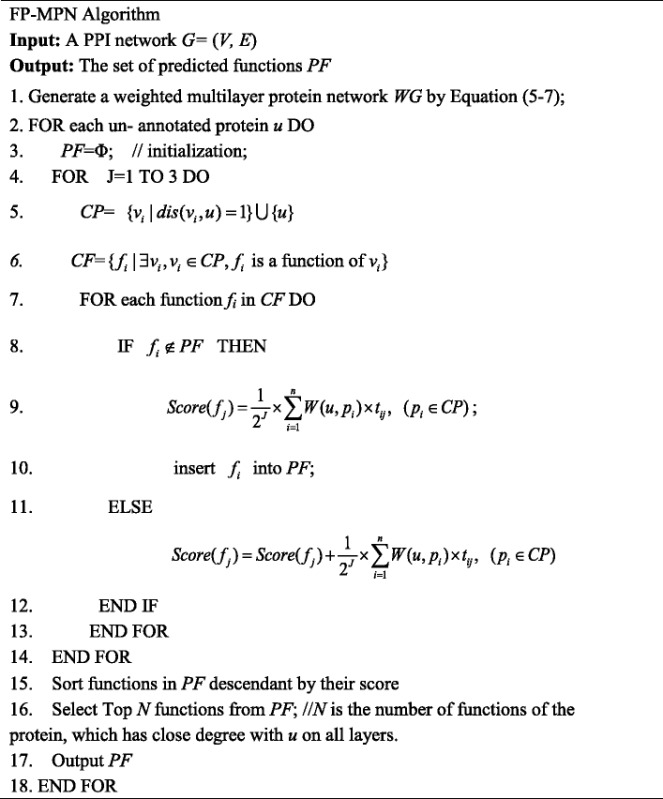


## Results and discussion

### Experimental data

The *S. cerevisiae* (yeast) PPI networks are widely used in the research of network-based function prediction methods, because the species of yeast has been well characterized by knockout experiments and is the most complete and convincible. Here, we also adopt the yeast PPI network to test our method. We have applied our method and four other competing algorithms by integrating network topological features, domain information, and protein complexes data: Zhang [15], DCS [16], domain combination similarity in context of protein complexes (DSCP) [16], and PON [17] on DIP data [29]. DSCP is a variant of DSC, which combines protein complex information. The DIP dataset, updated to Oct. 1, 2014, consists of 5017 proteins and 23,115 interactions among the proteins. The self-interactions and the repeated interactions are filtered out in DIP data. The annotation data of proteins used for method validation is the latest version (2012.3.3) downloaded from GO official website [30]. The GO system consists of three separate categories of annotations, namely molecular function (MF), biological process (BP), and cellular component (CC). The predictions are validated separately for each of the three GO categories. To avoid too special or too general, only those GO terms that annotate at least 10 and at most 200 proteins will be kept in the experiments. After processing by this step, the number of GO terms is 267. The domain data is derived from Pfam database [31], including 1107 different types of domains among 3056 proteins. As for the protein complex information, we used the dataset CYC2008 [32], which consists of 408 protein complexes involving 1492 proteins in the yeast PPI network. The GO data and Pfam domain data are transformed to use the ensemble genome protein entries because the original PPI network uses such a labeling system.

### Effect of access sequence of each layer

The access sequence of each layer in the MPN plays an important role in the performance of the proposed FP-MPN method. In this paper, the priority of each layer was determined using statistical analysis. Different schemes were used to sequence layers of the MPN and then compare these results to verify the effectiveness of the FP-MPN method. Table 3 depicts the results of FP-MPN when different schemes were adopted. Table 3 demonstrates that the first scheme (SCL → SDL → PIL), in which SCL was visited first and the SDL was visited second, performed the highest in terms of BP (biological process), MF (molecular function), and CC (cellular component). The comparison of these results with the statistical results show they are in agreement. Experimental results also verify the method used to access the sequence of each layer in the FP-MPN.

**Table 3:** The influence of access sequence

Categories	Schemes	Precision	Recall	*F*-measure	CR
BP	SCL → SDL → PIL	0.444	0.427	0.435	0.426
	SCL → PIL → SDL	0.462	0.401	0.429	0.374
	SDL → PIL → SCL	0.452	0.404	0.426	0.396
	SDL → SCL → PIL	0.442	0.424	0.433	0.422
	PIL → SDL → SCL	0.453	0.404	0.427	0.397
	PIL → SCL → SDL	0.459	0.398	0.426	0.372
MF	SCL → SDL → PIL	0.569	0.544	0.556	0.508
	SCL → PIL → SDL	0.566	0.535	0.55	0.495
	SDL → PIL → SCL	0.585	0.54	0.561	0.505
	SDL → SCL → PIL	0.568	0.543	0.555	0.507
	PIL → SDL → SCL	0.584	0.539	0.561	0.504
	PIL → SCL → SDL	0.573	0.541	0.557	0.5
CC	SCL → SDL → PIL	0.463	0.439	0.451	0.415
	SCL → PIL → SDL	0.468	0.43	0.448	0.4
	SDL → PIL → SCL	0.473	0.424	0.447	0.402
	SDL → SCL → PIL	0.461	0.439	0.45	0.413
	PIL → SDL → SCL	0.473	0.424	0.448	0.403
	PIL → SCL → SDL	0.467	0.429	0.447	0.4

### Leave-one-out cross-validation

A representative set of function prediction algorithms was run: FP-MPN, Zhang, DCS, DSCP, and PON, and their performance was examined using the leave-one-out cross-validation method. In the DIP PPI network, 2870, 1592, and 2427 proteins from a total of 5017 proteins were annotated by BP, MF, and CC, respectively. We analyzed the overall prediction performance of FP-MPN on these annotated proteins, as well as four other methods. The results are shown in Table 4, which include the average Precision, Recall, and *F*-measure and coverage rate (CR) of the various algorithms.

**Table 4:** Overall comparisons of various methods

Categories	Methods	MP	Precision	Recall	*F*-measure	CR
BP	FP-MPN	1595	0.444	0.427	0.435	0.426
	Zhang	810	0.225	0.220	0.222	0.216
	DCS	1148	0.312	0.314	0.313	0.327
	DSCP	1298	0.357	0.359	0.358	0.363
	PON	572	0.150	0.140	0.145	0.161
MF	FP-MPN	995	0.569	0.544	0.556	0.508
	Zhang	608	0.332	0.332	0.332	0.316
	DCS	839	0.461	0.462	0.461	0.441
	DSCP	927	0.518	0.515	0.516	0.489
	PON	413	0.223	0.216	0.22	0.228
CC	FP-MPN	1265	0.463	0.439	0.451	0.415
	Zhang	561	0.197	0.196	0.197	0.198
	DCS	876	0.306	0.309	0.307	0.315
	DSCP	1014	0.364	0.363	0.364	0.356
	PON	440	0.148	0.138	0.143	0.158

In Table 4, MP is the number of proteins which have been matched to at least one function with known function. Among the five methods, FP-MPN and PON are two methods of selecting top-ranking functions from the set of candidate functions, whereas the methods of Zhang, DCS, and DSCP are three methods of endowing un-annotated proteins with all functions of proteins with the highest similarity values. From Table 4, we can see that FP-MPN can predict functions for more proteins and archive higher performance than the other four methods, with respect to BP, MF, and CC. For BP, the *F*-measure of FP-MPN is 95.95, 38.98, 21.51, and 200 % higher than Zhang, DCS, DSCP, and PON, respectively. After integrating protein complexes and domains, DSCP improves the performance compared to DCS. FP-MPN outperforms DSCP, including the *F*-measure and coverage rate. When looking at MF, the performances of these five methods are better. The *F*-measure of FP-MPN is 67.47, 20.61, 7.75, and 152.73 % higher than the results using the methods of Zhang, DCS, DSCP, and PON, respectively. As for CC, the *F*-measure of FP-MPN is 128.93, 46.91, 23.9, and 215.38 % higher than the results using the methods of Zhang, DCS, DSCP, and PON, respectively. Compared to BP and MF, FP-MPN had a higher *F*-measure growth rate compared to other methods.

**Fig. 6:**
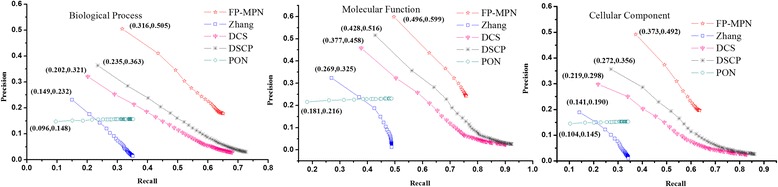
The precision-recall curves of FP-MPN compared to other four existing algorithms

A comprehensive comparison of the performances of these five methods was undertaken using a Precision-Recall (PR) curve to evaluate the global performance of every method in terms of the different strategies of function selection adopted by the five prediction methods. The same number of functions was chosen for each method, i.e., the top *K* functions of each prediction method. When examining the methods of Zhang, DCS, and DSCP, the top *M* (*M* < =*K*) proteins which had the highest similarity value were selected and the top *K* functions from the function list as a predictor of functions was listed in descending order according to the maximum value of protein similarity (e.g., given a certain function *F*_*i*_ found in more than one protein, the score of *F*_*i*_ is the similarity value of this protein when compared to the tested proteins). As for the FP-MPN and PON methods, the top *K* GO terms are chosen to assign functional properties to the unknown proteins (*K* ranges from 1 to 50). The areas under the curve (AUC) for FP-MPN and other methods are used to compare their performance. AUC is considered to be a standard method to assess the accuracy of predictive distribution models. From Fig. 6, we can see that FP-MPN outperforms other methods in terms of BP, MF, and CC. For example, on the BP, the AUC of FP-MPN is 347.67, 53.76, 31.76, and 195.46 % higher than Zhang, DCS, DSCP, and PON, respectively.

**Fig. 7:**
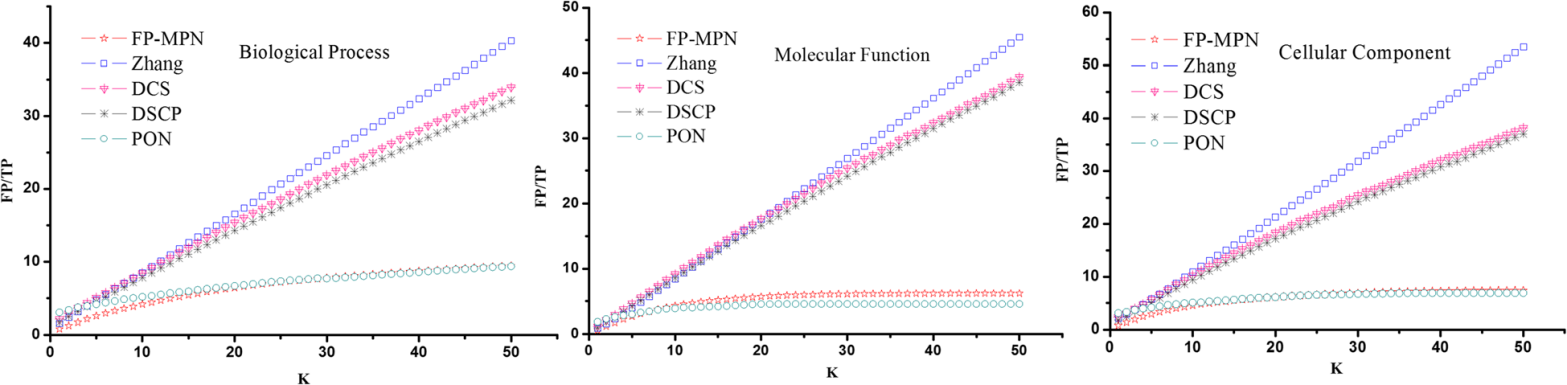
FP/TP curves of various methods

The number of incorrect predicted functions when matching a function correctly using these methods was determined. For each testing protein, the top *K* functions are selected as its predicted ones, and TP and FP values are calculated according to its known functions. The TP and FP values of all testing proteins are added to calculated TP and FP pairs. Selecting different values of *K* (ranging from 1 to 50), a FP/TP curve can be generated with different TP and FP pairs, as shown in Fig. 7. Figure 7 clearly shows that the curvature of FP-MPN curve is the lowest as compared to others, which means that, if matched functions are the same, the number of functions incorrectly matched by FP-MPN is the least. Table 5 lists the statistical results of the various FP/TP curves, including maximum value, the minimum value, the average value, and the middle value. These results indicates that to match a protein function correctly, the number of average noise functions (i.e., predicted function incorrectly matched) produced by FP-MPN is smaller compared to the Zhang, DCS, and DSCP methods. FP-MPN has comparable results with PON’s. For example, on the BP, the number of average noise functions of the methods of FP-MPN, Zhang, DCS, DSCP, and PON is 7, 21, 19, 18, and 7, respectively. The results illustrate that FP-MPN has the high prediction efficiency and accuracy.

**Table 5:** Statistical analysis of FP/TP of various methods

Categories	Methods	Maximum	Minimum	Average	Middle
BP	FP-MPN	9.44	0.72	6.48	7.18
	Zhang	40.29	1.59	20.96	21.04
	DCS	33.94	2.12	18.64	18.94
	DSCP	32.14	1.75	17.49	17.75
	PON	9.39	3.07	6.98	7.41
MF	FP-MPN	6.19	0.53	5.23	5.99
	Zhang	45.5	0.9	22.81	22.71
	DCS	39.41	1.18	21.28	21.88
	DSCP	38.54	0.94	20.4	20.73
	PON	4.57	1.85	4.2	4.57
CC	FP-MPN	7.39	0.72	5.88	6.59
	Zhang	53.51	2.12	27.29	27.09
	DCS	38.15	2.36	21.49	22.25
	DSCP	37.02	1.81	20.45	21.04
	PON	6.88	3.07	6.02	6.57

### Tenfold cross-validation

**Fig. 8:**
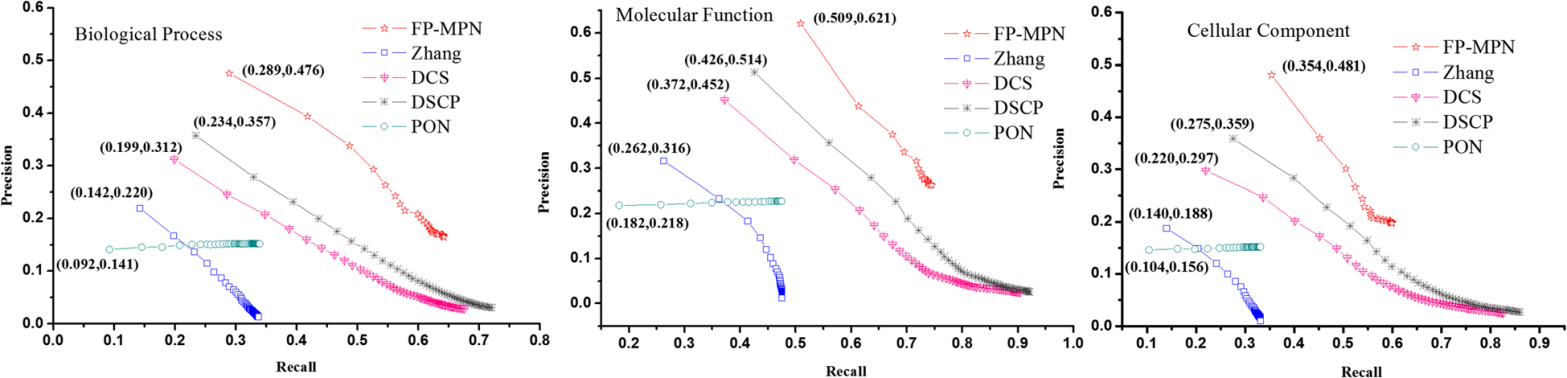
The precision-recall curves of various methods using tenfold cross-validation

The performance of FP-MPN was tested using leave-one-out validation. Experimental results demonstrate improvements when predicting protein functions by the FP-MPN method compared to competing methods. However, in practical applications, there are much more proteins without annotations, instead of one unknown protein. In this section, we will use the leave-percent-out cross-validation method to verify the effectiveness of FP-MPN on PPI networks that have less functional information. Tenfold cross-validation is a widely used leave-percent-out cross-validation, which is used in this paper. The tenfold cross-validation requires the entire set of examples to be divided into ten equal sets randomly. Nine of the ten parts are used for training, and one part is used for testing. This is repeated ten times, each time using another testing set. We evaluate the performance of each method using area under precision-recall (PR) curve. Figure 8 illustrates the PR curve using tenfold cross-validation, in terms of biological processes, molecular functions, and cellular components. When compared to the results of leave-one-out cross-validation, the performance of all methods using tenfold cross-validation decrease slightly, due to the decrease of the number of training proteins. It appears that Fig. 8 is very similar to Fig. 6, except for the coordinate values of the various methods. Figure 8 demonstrates that FP-MPN still outperforms other methods when tenfold cross-validation is used to test all methods.

### Analysis of the overlaps and differences between FP-MPN and other methods

To further analyze the differences between the FP-MPN and other methods, we selected 12 testing proteins and predicted their functions using the five methods. Table 6 lists the functions of these selected proteins predicted by various methods. The third to the seventh column of Table 6 lists functions predicted by the FP-MPN, Zhang, DCS, DSCP, and PON methods, respectively. In this table, functions in italics represent the matched functions of the testing proteins, the rest are mismatched functions. In Table 6, we can see that FP-MPN can record more correct functions and fewer error functions compared to the other competing methods.

**Table 6:** Selected functions predicted by various methods

Categories	Proteins	FP-MPN	Zhang	DCS	DSCP	PON
BP	YGL100W (8 GO terms)	*GO:0006409* *GO:0006607* *GO:0006913* *GO:0006999* *GO:0006406* *GO:0006609* *GO:0006611* *GO:0006407* GO:0000973 GO:0000055	GO:0000723 GO:0006348 GO:0006355 GO:0051568	GO:0043161	GO:0043161	GO:0000001 GO:0000002 GO:0000027 GO:0000055 GO:0000082 GO:0000086 GO:0000122 GO:0000209
	YNL262W (7 GO terms)	*GO:0006272* *GO:0006273* *GO:0006289* *GO:0006298* GO:0000084	*GO:0006273* GO:0000084 GO:0006270	*GO:0006273* GO:0000084 GO:0006270	*GO:0006273* GO:0000084 GO:0006270	*GO:0006272* *GO:0006273* *GO:0006289* GO:0000084 GO:0006260 GO:0006270 GO:0006284
	YLR321C (6 GO terms)	*GO:0006337* *GO:0006368* *GO:0043044* *GO:0000086*	*GO:0006302* *GO:0043044* GO:0006338 GO:0042766 GO:0045944	*GO:0006302* *GO:0043044* GO:0006338 GO:0042766 GO:0045944	*GO:0006302* *GO:0043044* GO:0006338 GO:0042766 GO:0045944	*GO:0006302* *GO:0043044* GO:0006338 GO:0042766 GO:0045944
	YBR278W (5 GO terms)	*GO:0006272* *GO:0006273* *GO:0006289* *GO:0006298* *GO:0006348* GO:0006303 GO:0007064		*GO:0006348* GO:0000723 GO:0006281 GO:0007064 GO:0030466	*GO:0006348* GO:0000723 GO:0006281 GO:0007064 GO:0030466	
MF	YBR114W (3 GO terms)	*GO:0004842* *GO:0003684* *GO:0008094*	*GO:0008094*	*GO:0008094*	*GO:0008094*	GO:0000386 GO:0000990 GO:0001102
	YJR052W (3 GO terms)	*GO:0004842* *GO:0003684* *GO:0008094*		GO:0008134	GO:0043130	
	YJR140C (3 GO terms)	*GO:0003677* *GO:0031491* *GO:0003714*		GO:0046933 GO:0046961	*GO:0003677* *GO:0031491*	
	YBL021C (2 GO terms)	*GO:0001077* *GO:0000978*	GO:0003713 GO:0003714	GO:0003713 GO:0003714	GO:0003713 GO:0003714	GO:0003713 GO:0003714
CC	YNL161W (6 GO terms)	*GO:0005933* *GO:0005934* *GO:0005935* *GO:0043332*	*GO:0005935* GO:0005816	*GO:0005935* GO:0005816	*GO:0005935* GO:0005816	*GO:0000131* GO:0000139 GO:0000142 GO:0000307 GO:0000324 GO:0000329
	YBR198C (3 GO terms)	*GO:0000124* *GO:0046695* *GO:0005669*	GO:0070210	GO:0070210	GO:0070210	*GO:0000124* GO:0000139 GO:0000228
	YDR167W (3 GO terms)	*GO:0000124* *GO:0046695* *GO:0005669*		GO:0005666	*GO:0000124* *GO:0046695*	
	YNL273W (3 GO terms)	*GO:0031298* *GO:0000228* *GO:0043596*		GO:0005751	GO:0005751	

In addition, we continued to look for sources of functions predicted by various methods. For the protein YGL100W, the functions set predicted by the method of Zhang consists of GO:0000723, GO:0006348, GO:0006355, and GO:0051568, which were derived from the protein YAR003W. In this study, YAR003W is regarded as having the most similar domain to YGL100W among all the proteins. Unfortunately, these predicted functions are mismatched by the real functions of YGL100W. As for DCS and DSCP, the protein YCL039W is considered to be the most similar in domain to YGL100W than the other known proteins. Similarly, the predicted functions of GO:0043161, which were derived from YCL039W, created errors in predicted functions for YAR003W. Predicted functions by PON were GO:0000001, GO:0000002, GO:0000027, GO:0000055, GO:0000082, GO:0000086, GO:0000122, and GO:0000209, which were derived from YBR234C, YJL112W, YKL021C, YDR267C, YDR364C, YFL009W, YLR055C, and YIL046W, respectively. All of these proteins have at least one domain with YGL100W. So, we can draw a conclusion that we cannot predict functions for the protein YGL100W based on domain information only. Our FP-MPN predicts ten functions, in which eight are matched and two are mismatched. These matched functions were derived from protein YDL116W, which is located in the transcription factor TFIID complex with the YGL100W protein. FP-MPN successfully matched eight functions for the protein YGL100W, with the help of protein complexes information. The results suggest that complexes information improves the accuracy of protein function prediction. However, protein complexes data is also used in the DSCP methods, which has a different predictor results compared to that of FP-MPN. This could be due to the difference in how the data is used between the two methods. For the protein YNL262W, the methods of Zhang, DCS, and DSCP created the same function lists, consisting of GO:0006273, GO:0000084, and GO:0006270. These three functions are derived from the protein YNL102W, which has common domains with the protein YNL262W. In the predicted functions list, only GO:0006273 is correct as a function for the protein YNL102W. Compared to the methods of Zhang, DCS, and DSCP, PON can identify two other correct functions GO:0006273 and GO:0006289 from another protein YDL102W, which shares domains with the protein YNL102W. The result suggests that annotating proteins according to multiple known proteins is more reliable than predicting functions from a single protein. Besides the three matched functions identified by other methods, FP-MPN identifies a new correct function GO:0006298. In this example, FP-MPN predicts more matched functions compared to other methods, due to the domain and complexes information being used. This phenomenon suggests that proper use of multiple heterogeneous biological data can effectively improve the performance of function prediction algorithms. The analysis for the rest of the ten proteins described above is consistent with that of YGL100W and YNL262W.

### Efficiency analysis

**Fig. 9:**
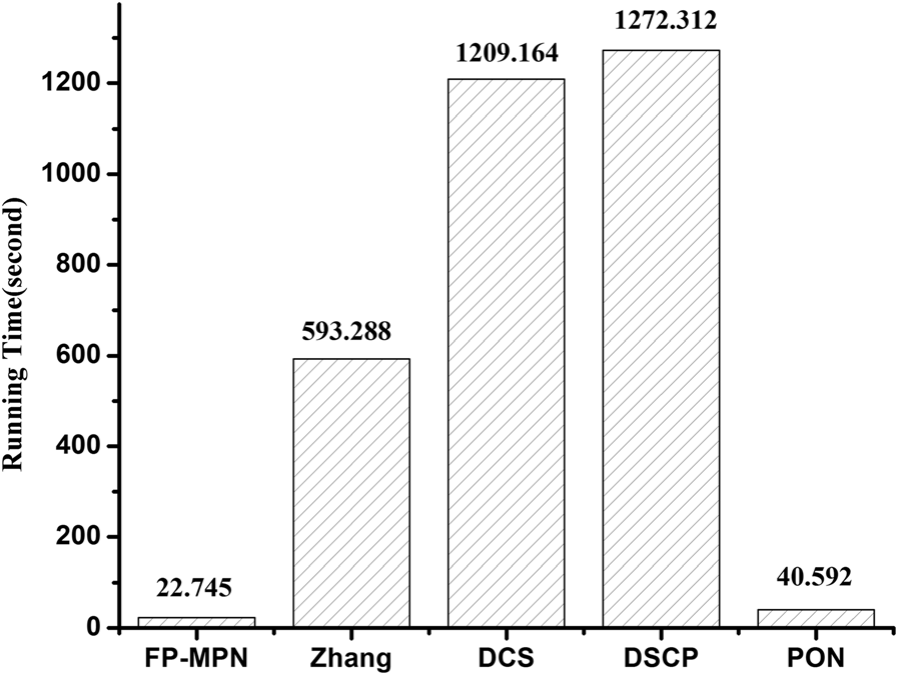
Comparison of the running time of various methods

To compare the efficiency of these methods, we ran FP-MPN and competing methods under the same conditions and looked at their running time. All methods in this paper were run on a notebook computer with Inter(R) Core(TM) i5-4300M 2.6 GHz CPU and 4 GB RAM. Figure 9 illustrates a comparison of the running time of FP-MPN and the other four methods used for predicting protein functions. The methods of Zhang, DCS, and DSCP are all based on combined number computation. So, they have the disadvantage of being time consuming. From Fig. 9, it can be seen that FP-MPN is extremely fast, 25, 52, 55, and 0.8 times faster than the methods of Zhang, DCS, DSCP, and PON, respectively. As protein-protein interactions are accumulating, FP-MPN can be used in larger scale PPI networks.

## Conclusions

Different types of interactions or connections play different roles in protein function prediction. Combining multiple interactions or connections between two proteins could reduce the impact of false negatives and increase the number of correct predicted functions. However, there appears to be more false functions identified compared to positive functions, thus the overall performance of function prediction would not be improved greatly. In this paper, multilayer protein networks (MPN) are constructed based on topological characteristics, protein domain information, and protein complex information, with each layer given various priorities. Based on the constructed networks, we proposed a new method, named FP-MPN, to predict the functions of a particular protein. The proposed method is based around visiting each layer of the MPN in turn and forming a set of candidate neighbors with known functions. The set of predicted functions is then formed and all of these functions are scored and sorted. Each layer contributes differently to the predicted functions in the un-annotated protein. The experimental results indicate that it is an effective method to predict protein functions.

## Additional file

Additional file 1: Supplementary Data are available, including Complexes.txt: The CYC2008 dataset, which consists of 408 protein complexes involving 1492 proteins in the yeast PPI network. DIP141001.txt: The DIP dataset, updated to Oct.1, 2014, consists of 5017 proteins and 23115 interactions among the proteins. Domain.txt: The domain data derived from Pfam database, including 1107 different types of domains among 3056 proteins. FP-MPN.exe: The FP-MPN algorithm. FPMPN.txt: The predicted results by FP-MPN. GO_C.txt, GO_F.txt and GO_P.txt: Represents cellular component (CC), molecular function (MF) and biological process (BP), respectively. (ZIP 437 kb)
